# Association of pulmonary vein isolation and major cardiovascular events in patients with atrial fibrillation

**DOI:** 10.1007/s00392-022-02015-0

**Published:** 2022-04-11

**Authors:** Marc Girod, Michael Coslovsky, Stefanie Aeschbacher, Christian Sticherling, Tobias Reichlin, Laurent Roten, Nicolas Rodondi, Peter Ammann, Angelo Auricchio, Giorgio Moschovitis, Richard Kobza, Patrick Badertscher, Sven Knecht, Philipp Krisai, Andrea Marugg, Helena Aebersold, Elisa Hennings, Miquel Serra-Burriel, Matthias Schwenkglenks, Christine S. Zuern, Leo H Bonati, David Conen, Stefan Osswald, Michael Kühne

**Affiliations:** 1 Cardiovascular Research Institute Basel, University Hospital Basel, University of Basel, Basel, Switzerland; 2 Department of Cardiology, Department of Medicine, University Hospital Basel, University of Basel, Basel, Switzerland; 3 Clinical Trial Unit Basel, Department of Clinical Research, University Hospital Basel, Basel, Switzerland; 4 Department of Cardiology, Inselspital, Bern University Hospital, University of Bern, Bern, Switzerland; 5 Institute of Primary Health Care (BIHAM), University of Bern, Bern, Switzerland; 6 Department of General Medicine, Inselspital, Bern University Hospital, University of Bern, Bern, Switzerland; 7 Department of Cardiology, Kantonsspital St. Gallen, St. Gallen, Switzerland; 8 Division of Cardiology, Institute Cardiocentro Ticino, Lugano, Switzerland; 9 Division of Cardiology, Ente Ospedaliero Cantonale, Regional Hospital of Lugano, Lugano, Switzerland; 10 Department of Cardiology, Luzerner Kantonsspital, Lucerne, Switzerland; 11 Epidemiology, Biostatistics, and Prevention Institute, University of Zürich, Zürich, Switzerland; 12 Institute of Pharmaceutical Medicine, University of Basel, Basel, Switzerland; 13 Department of Neurology and Stroke Center, University Hospital Basel, University of Basel, Basel, Switzerland; 14 Population Health Research Institute, McMaster University, Hamilton, Canada

**Keywords:** Atrial fibrillation, Pulmonary vein isolation, Adverse outcome events, Coarsened exact matching

## Abstract

**Background:**

Patients with atrial fibrillation (AF) face an increased risk of adverse cardiovascular events. Evidence suggests that early rhythm control including AF ablation may reduce this risk.

**Methods:**

To compare the risks for cardiovascular events in AF patients with and without pulmonary vein isolation (PVI), we analysed data from two prospective cohort studies in Switzerland (*n* = 3968). A total of 325 patients who had undergone PVI during a 1-year observational period were assigned to the PVI group. Using coarsened exact matching, 2193 patients were assigned to the non-PVI group. Outcomes were all-cause mortality, hospital admission for acute heart failure, a composite of stroke, transient ischemic attack and systemic embolism (Stroke/TIA/SE), myocardial infarction (MI), and bleedings. We calculated multivariable adjusted Cox proportional-hazards models.

**Results:**

Overall, 2518 patients were included, median age was 66 years [IQR 61.0, 71.0], 25.8% were female. After a median follow-up time of 3.9 years, fewer patients in the PVI group died from any cause (incidence per 100 patient-years 0.64 versus 1.87, HR 0.39, 95%CI 0.19–0.79, *p* = 0.009) or were admitted to hospital for acute heart failure (incidence per 100 patient-years 0.52 versus 1.72, HR 0.44, 95%CI 0.21–0.95, *p* = 0.035). There was no significant association between PVI and Stroke/TIA/SE (HR 0.94, 95%CI 0.52–1.69, *p* = 0.80), MI (HR 0.43, 95%CI 0.11–1.63, *p* = 0.20) or bleeding (HR 0.75, 95% CI 0.50–1.12, *p* = 0.20).

**Conclusions:**

In our matched comparison, patients in the PVI group had a lower incidence rate of all-cause mortality and hospital admission for acute heart failure compared to the non-PVI group.

**ClinicalTrials.gov Identifier:**

NCT02105844, April 7th 2014.

**Graphical abstract:**

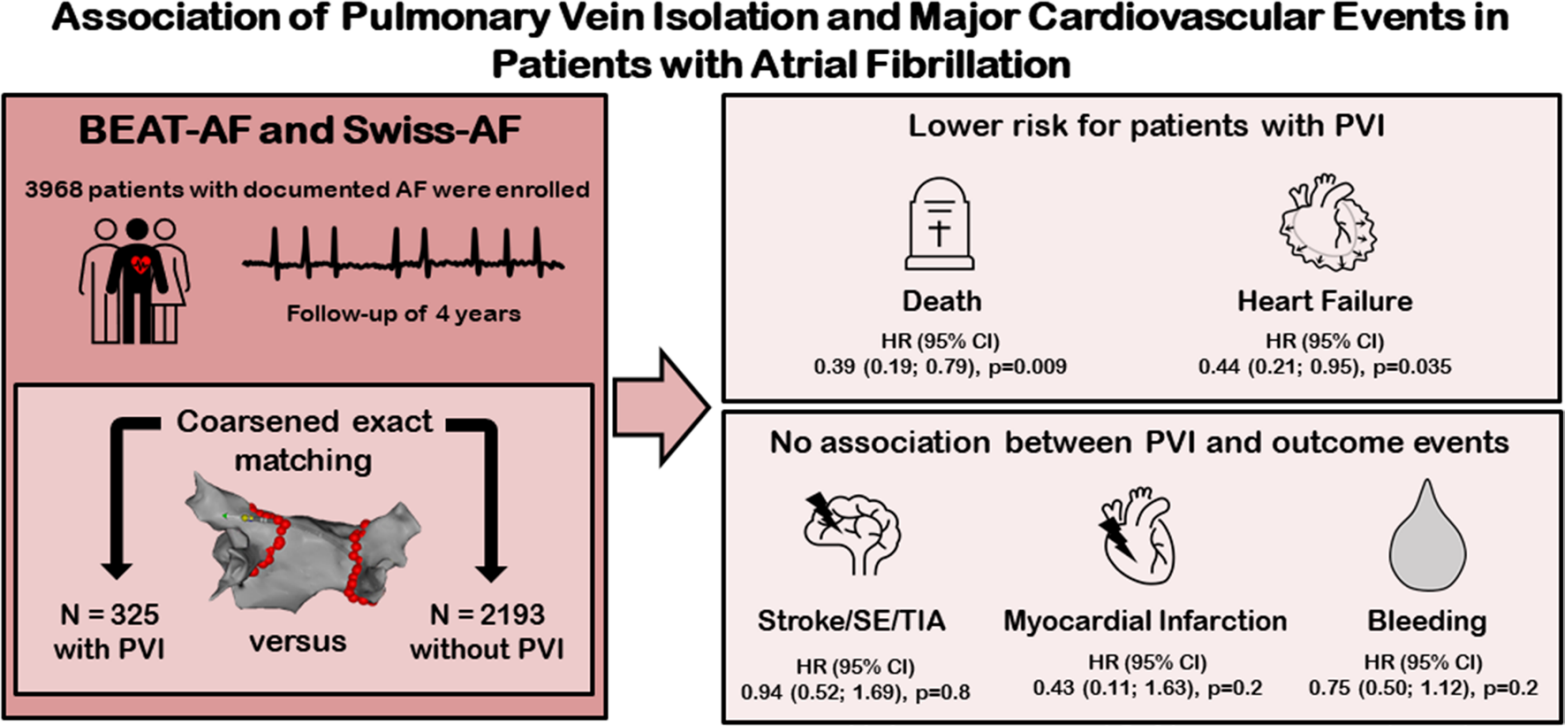

**Supplementary Information:**

The online version contains supplementary material available at 10.1007/s00392-022-02015-0.

## Introduction

Even in well-treated patients, atrial fibrillation (AF) is strongly associated with an increased risk of death, congestive heart failure, stroke and cognitive dysfunction [1–6]. Whereas rate control medication may relieve symptoms in AF patients, several older trials comparing rate and rhythm control treatment did not show superiority of medical rhythm control in terms of death and major cardiovascular events, both in AF patients with and without heart failure [7–9].

Catheter ablation has been shown to be more effective than medical rhythm control in reducing the burden of AF [10–12]. Studies also suggested that catheter ablation improves left ventricular systolic function and reduces hospitalizations due to heart failure [5, 13–15]. The CABANA trial, in turn, reported no significant reduction in death, disabling stroke, serious bleeding, or cardiac arrest among patients receiving catheter ablation, but a large number of crossovers occurred [16]. The recent multicentre randomized EAST-AFNET 4 trial compared early rhythm control (mostly using antiarrhythmic drugs) to usual care (e.g. rate-control therapy) among patients with a recent diagnosis of AF (< 12 months) [17]. The trial reported a lower risk of cardiovascular death or stroke in the early rhythm control group. However, most of the patients were initially treated with anti-arrhythmic drugs and only a minority of patients (19.4%) had an AF ablation after 2 years of follow-up.

In this context, we aimed to investigate the association of pulmonary vein isolation (PVI) with all-cause mortality, hospital admission for acute heart failure, myocardial infarction (MI), stroke, and bleeding in two large contemporary real-world cohorts of AF patients, the Basel Atrial Fibrillation Cohort (BEAT-AF) and Swiss Atrial Fibrillation Cohort (Swiss-AF).

## Methods

### Study design and patient population

For this analysis, we combined data of the BEAT-AF and Swiss-AF studies. Both are large prospective, observational, multicentre cohort studies in Switzerland. The detailed methodology has been described previously [18]. Across seven centres in Switzerland, 1553 patients with documented AF were enrolled in the BEAT-AF study between 2010 and 2014. Recruitment for the Swiss-AF cohort took place between 2014 and 2017. 2415 patients with documented AF were enrolled in Swiss-AF across 14 centres in Switzerland. The main inclusion criterion for both cohorts was documented AF. Patients with short secondary, reversible episodes of AF (e.g., after cardiac surgery) or patients who were unable to sign informed consent were not enrolled. Individuals with an acute illness within the last four weeks could be enrolled once the episode had resolved. Patients enrolled in BEAT-AF were not eligible to participate in Swiss-AF.

From a total of 3968 patients, we excluded 361 patients: seven patients from Beat-AF due to accidental enrolment in Swiss-AF, 313 for missing information on PVI status, and 41 due to missing follow-up information, resulting in 3607 patients included in this analysis (Fig. S1). The study protocol was approved by the main Ethics committee EKNZ (Ethikkommission Nordwest- und Zentralschweiz, 2021-00701; PB_2016-01182). Informed written consent was obtained from each participant.

### Data collection

In both cohorts, information on individual patient characteristics, medical history including adverse clinical outcome events, risk factors, current medication and history of arrhythmia-related interventions including PVI were collected using standardized case report forms (CRF). Body mass index (BMI) was calculated as weight in kilograms divided by height in meters squared. Smoking status was categorized in current, past or never smokers. Blood pressure was measured three times in a supine position and the mean of all values was used. AF type was categorized as paroxysmal, persistent and permanent according to the 2010 AF guidelines of the European Society of Cardiology [19]. The collected data were updated during yearly follow-up visits and telephone follow-ups.

### Assessment of PVI status

Patients were separated in a PVI and non-PVI group. A total of 325 patients had undergone PVI (76% radiofrequency ablation, 24% cryoballoon ablation) during the first year of follow-up and were assigned to the PVI group. No additional lesions (e.g. linear lesions) in addition to PVI were performed. In each case, the procedure was confirmed by a medical report. Patients with no PVI during this time were assigned to the control group (non-PVI group). To establish a non-PVI group without any history of PVI, 673 patients were excluded due to a registered PVI prior to study enrolment, resulting in 2609 patients without a history of PVI in the control group, and a total of 2934 patients eligible for matching (Fig. S1).

### Outcome events

Main outcomes included all-cause mortality, hospital admission for acute heart failure, a composite of stroke, transient ischemic attack and systemic embolism (Stroke/TIA/SE), myocardial infarction (MI), and a composite of major and clinically relevant non-major bleedings. Additionally, we included a composite endpoint consisting of death from cardiovascular causes, stroke, or hospital admission for acute heart failure or myocardial infarction (Death/Stroke/aHF/MI), modelled after the EAST-AFNET 4 trial. All reported outcome events were independently validated by two physicians according to the same BEAT-AF or Swiss-AF outcome definitions provided in Table S1. In case of discrepancy, a third physician was consulted.

### Statistical analysis

Baseline characteristics were stratified by the presence or absence of PVI between baseline and 1-year follow-up. For this analysis, data of the 1-year follow-up visit were set as the new baseline. Values were presented as means (± standard deviation), medians (interquartile range) or counts (percentages), as appropriate. Groups were compared using Chi-square test for categorical variables and Student’s *t*-test or Mann–Whitney-*U*-test for continuous variables. The standardized mean difference (SMD) was calculated by the difference in means between the two groups divided by the standard deviation. A detailed illustration of the SMD for each baseline characteristic is portrayed in the supplement (Fig. S2).

To investigate the association of PVI with adverse outcome events, patients of the PVI group were matched to controls of the non-PVI group. Matching was based on age, sex, AF type, history of diabetes and history of hypertension using Coarsened Exact Matching (CEM) [20]. Age was divided into four groups based on quartiles, and AF type was categorized as paroxysmal or non-paroxysmal. All observations were sorted into strata, each of which had identical values for all matching covariates. By means of CEM, 416 patients out of the non-PVI group could not be matched in a stratum with at least one corresponding PVI observation, leaving 2518 patients for the final analysis (Fig. S1).

Person-years for each outcome event were calculated from baseline until death, withdrawal, loss to follow-up, last completed visit, first occurrence of the respective outcome event or date of first PVI after the observational period for patients in the non-PVI group. Cumulative incidence curves were plotted for every outcome event stratified by PVI status (Figs. S3.1––S3.6).

We used Cox proportional-hazard models to further investigate the associations between PVI and clinical outcome events. The first model was adjusted for age within each stratum. The second model was additionally adjusted for history of heart failure and coronary artery disease, given that we did not match for these important confounders in the first place. Results are presented as hazard ratios (HR) and 95% confidence intervals (CI). Events prior to the newly set baseline have not been accounted for in these analyses. Cox proportional-hazard assumptions were checked using Schoenfeld residuals and cloglog plots. To account for the effect of different strata sizes, weights generated throughout the matching process were applied during the analyses.

To address the potential effect of subcohort selection, we conducted a sensitivity analysis without excluding patients with a prior PVI before enrolment and without defining a matched population within our BEAT-AF and Swiss-AF cohort (Fig. S4). For the sensitivity analysis, data of the study enrolment were set as the baseline. We constructed two Cox proportional-hazards models to investigate the association between PVI and the outcome events. To take account of potential changes in PVI status and other covariates, variables were updated over time. The first model was adjusted for age and sex. The second model was additionally adjusted for AF type, history of hypertension, diabetes, coronary artery disease and heart failure hospitalizations.

All statistical analyses were performed using R version 4.0.3 (R Foundation for statistical computing, Vienna, Austria). The matching was completed using the cem package version 1.1.27 [20].

## Results

Baseline characteristics of our matched sample stratified by PVI during the observation period are presented in Table 1. The median age of the cohort was 66.0 years [IQR 61.0, 71.0], and 651 patients (25.8%) were female. The median time since AF diagnosis was 3.5 years [IQR 1.8, 7.2]. Overall, 1550 (61.6%) patients had paroxysmal AF. 1808 (72.3%) were on oral anticoagulation, 1690 (67.1%) took a betablocker, and 688 (27.3%) were on an antiarrhythmic drug (class Ic or III).

**Table 1:** Baseline Characteristics stratified by PVI status in the matched population

Characteristic	Overall	Non-PVI group	PVI group
*n*	2518	2193	325
Age, y (median [IQR])	66.0 [61.0, 71.0]	67.0 [61.0, 71.0]	65.0 [57.0, 71.0]
Female sex, *n* (%)	651 (25.8)	567 (25.8)	84 (25.8)
BMI, kg/m^2^ (median [IQR])	27.0 [24.3, 30.4]	27.1 [24.2, 30.5]	26.6 [24.5, 29.7]
Active smoker, *n* (%)	246 (9.8)	218 (9.9)	28 (8.6)
Education level, *n* (%)			
Basic	249 (9.9)	223 (10.2)	26 (8.0)
Middle	1212 (48.1)	1080 (49.2)	132 (40.7)
Advanced	1043 (41.4)	877 (40.0)	166 (51.2)
AF type, *n* (%)			
Paroxysmal	1549 (61.5)	1349 (61.5)	200 (61.5)
Non-paroxysmal	969 (38.5)	844 (38.5)	125 (38.5)
Time since AF diagnosis, y [median (IQR)]	3.5 [1.8, 7.2]	3.5 [1.8, 7.1]	4.2 [1.9, 7.9]
Medical history, *n* (%)			
Stroke or TIA	369 (14.6)	339 (15.4)	30 (9.2)
Hypertension	1449 (57.5)	1262 (57.5)	187 (57.5)
Heart failure	519 (20.6)	476 (21.7)	43 (13.2)
Coronary artery disease	504 (20.0)	472 (21.5)	32 (9.8)
Myocardial infarction	290 (11.5)	278 (12.7)	12 (3.7)
Diabetes	163 (6.5)	142 (6.5)	21 (6.5)
Renal failure	279 (11.1)	256 (11.7)	23 (7.1)
RFA of atrial flutter	217 (8.6)	150 (6.8)	67 (20.6)
ECV	947 (37.6)	780 (35.6)	167 (51.4)
PTCA	387 (15.4)	361 (16.5)	26 (8.0)
CABG	162 (6.4)	157 (7.2)	5 (1.5)
Medication, *n* (%)			
Oral anticoagulation	1808 (71.8)	1566 (71.4)	242 (74.5)
Antiplatelet therapy	512 (20.3)	475 (21.6)	37 (11.4)
Antiarrhythmic drugs	688 (27.3)	611 (27.8)	77 (23.7)
Betablocker	1691 (67.2)	1488 (67.9)	203 (62.5)
CHA_2_DS_2_-VASc score (mean ± SD)	2.4 ± 1.6	2.4 ± 1.6	2.0 ± 1.4

Values presented as mean ± SD, median (interquartile range) or n (%).Matching was based on age categories, sex, AF type, history of diabetes and history of hypertension using Coarsened Exact Matching (CEM). To account for the effect of different strata sizes, weights generated throughout the matching process were applied. Patients with a history of PVI at baseline were not eligible for the control group

Missing values: Active smoker (*n* = 27), Education level (*n* = 14), Oral anticoagulation (*n* = 19), Antiplatelet therapy (*n* = 43)

*CHA*_*2*_*DS*_*2*_*-VASc* congestive heart failure, hypertension, age ≥ 75 years. (2 points), diabetes, prior stroke or TIA or thromboembolism (2 points), vascular disease, age 65–74 years, female sex; *IQR* interquartile range; *BMI* body mass index; *AF* atrial fibrillation; *TIA* transient ischemic attack; *RFA* radiofrequency ablation; *ECV* electrical cardioversion; *PTCA* percutaneous transluminal coronary angioplasty; *CABG* coronary artery bypass graft

Overall, 325 patients (12.9%) underwent PVI during the observational period. Compared to patients without PVI, patients in the PVI group were younger [65.0 years (IQR 57.0, 71.0) vs 67.0 years (IQR 61.0, 71.0)], had a lower CHA_2_DS_2_-VASc-Score (2.0 ± 1.4 vs 2.4 ± 1.6), and a lower prevalence of coronary artery disease, history of heart failure, diabetes, and renal failure. After CEM, in general the baseline characteristics were well distributed, resulting in a low standardized mean difference (SMD) between the PVI and non-PVI group. Nevertheless, moderate differences in potential confounders (history of radiofrequency ablation (RFA) of atrial flutter, history of electrical cardioversion, intake of antiplatelet therapy, history of coronary artery disease) remained (Fig. S2).

**Fig. 1:**
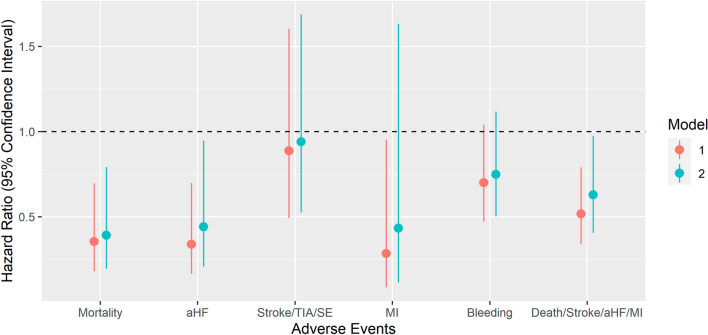
Multivariable adjusted cox-proportional hazards models for adverse events in a matched population. *Mortality* All-cause mortality; *aHF* Hospital admission for acute heart failure; *Stroke/TIA/SE* Stroke, transient ischemic attack, and systemic embolism;  *MI* Myocardial infarction; *Bleeding* Major bleeding and clinically relevant non-major bleeding, *Death/Stroke/aHF/MI* Composite of death from cardiovascular causes, stroke, or hospital admission for acute heart failure or myocardial infarction. The colours represent the two different models. Model 1 was adjusted for age, within each stratum with identical values for all matching covariates. Model 2 was additionally adjusted for history of coronary artery disease and heart failure hospitalization, within each stratum with identical values for all matching covariates

The absolute and relative risks are presented in Table 2 and Fig. 1. During a median follow-up time of 3.9 years, 173 patients died, 154 were admitted to the hospital for acute heart failure, 107 suffered a Stroke/TIA/SE, 53 had a MI, 298 presented with a bleeding, and the composite of Death/Stroke/aHF/MI occurred in 332 patients. Incidence rates per 100 patient-years in the PVI group and the non-PVI group were 0.64 versus 1.87 for all-cause mortality, 0.52 versus 1.72 for aHF, 0.91 versus 1.09 for Stroke/TIA/SE, 0.19 versus 0.58 for MI, 2.16 versus 3.27 for bleeding, and 1.72 versus 3.46 for Death/Stroke/aHF/MI. There was a significant association of PVI with all-cause mortality (HR 0.39, 95%CI 0.19–0.79, *p* = 0.009), aHF (HR 0.44, 95%CI 0.21–0.95, *p* = 0.035) and Death/Stroke/aHF/MI (HR 0.63, 95%CI 0.40–0.97, *p* = 0.038) in multivariable Cox models. There was no significant association with Stroke/TIA/SE (HR 0.94, 95%CI 0.52–1.69, *p* = 0.80), MI (HR 0.43, 95%CI 0.11–1.63, *p* = 0.20) or bleeding (HR 0.75, 95% CI 0.50–1.12, *p* = 0.20).

**Table 2:** Association between PVI and cardiovascular events in the matched population

	Group^a^	Number of events	Incidence rate (per 100 person years)	Model 1^c^		Model 2^b^	
				Hazard ratio (95% CI)	*p* value	Hazard ratio (95% CI)	*p* value
Adverse outcome events							
All-cause mortality	PVI	10	0.64	0.35 (0.18, 0.70)	0.003	0.39 (0.19, 0.79)	0.009
	Non-PVI	163	1.87				
Hospital admission for acute heart failure	PVI	8	0.52	0.34 (0.16, 0.70)	0.003	0.44 (0.21, 0.95)	0.035
	Non-PVI	146	1.72				
Stroke/TIA and systemic embolism	PVI	14	0.91	0.89 (0.49, 1.60)	0.7	0.94 (0.52, 1.69)	0.8
	Non-PVI	93	1.09				
Myocardial infarction	PVI	3	0.19	0.28 (0.08, 0.95)	0.041	0.43 (0.11, 1.63)	0.2
	Non-PVI	50	0.58				
Major and clinically relevant non-major bleeding	PVI	32	2.16	0.70 (0.47, 1.04)	0.078	0.75 (0.50, 1.12)	0.2
	Non-PVI	266	3.27				
Composite of death from cardiovascular causes, stroke, or hospital admission for acute heart failure or myocardial infarction	PVI	26	1.72	0.52 (0.34, 0.79)	0.002	0.63 (0.40, 0.97)	0.038
	Non-PVI	306	3.46				

^a^Matching was based on age categories, sex, AF type, history of diabetes and history of hypertension using Coarsened Exact Matching (CEM). To account for the effect of different strata sizes, weights generated throughout the matching process were applied. Patients with a history of PVI at baseline were not eligible for the control group

^b^Model 1 was adjusted for age, within each stratum

^c^Model 2 was additionally adjusted for history of coronary artery disease and heart failure, within each stratum

Baseline characteristics for the population used in our sensitivity analysis to address the potential effect of subcohort selection are presented in Table S2. Overall, 3885 patients were included in the sensitivity analysis. Median age of this patient cohort was 72.4 years [IQR 66.1, 78.1], and 1092 patients (28.1%) were female. Compared to the matched population of the main analysis, this population was older [72.4 years (IQR 66.1, 78.1) vs 66.0 years (IQR 61.0, 71.0)] and had a higher burden of cardiovascular risk factors and comorbidities.

The results from the sensitivity analysis were consistent with those of our main analysis in the matched population, as shown in Fig. S5. The absolute and relative risks of the outcomes are presented in Table S3. After a median follow-up time of 4.5 years, significantly fewer patients who underwent PVI during our study died from any cause (incidence per 100 patient-years 0.54 vs 3.46, HR 0.40, 95%CI 0.23–0.71, *p* = 0.002) or were admitted to hospital for acute heart failure (incidence per 100 patient-years 0.81 vs 3.56, HR 0.57, 95%CI 0.35–0.91, *p* = 0.019) compared to patients without a PVI. There was no significant association between PVI and Stroke/TIA/SE (HR 0.62, 95%CI 0.32–1.21, *p* = 0.2), MI (HR 0.36, 95%CI 0.13–1.00, *p* = 0.049) or bleeding (HR 0.79, 95% CI 0.58–1.07, *p* = 0.12).

## Discussion

In this matched comparison of patients with AF participating in two Swiss cohort studies, patients with a PVI in the previous year had a significantly lower rate of all-cause mortality and hospital admission for acute heart failure. We observed no significant association between PVI and Stroke/TIA/SE and MI. There was no significantly lower incidence in bleedings between the groups. The results were similar when different analysis techniques were used.

After uni- and multivariable adjustment, patients in the PVI group had a lower rate of all-cause mortality. These findings are consistent with a recent multicentre, randomized trial in heart failure patients [13], but they differ from those of the CABANA trial where only a minority of 15% had heart failure and no significant reduction in mortality was shown [16]. However, the CABANA trial reported lower-than-expected event rates and a large number of treatment crossovers, which may have affected the treatment effect. The recently published EAST-AFNET 4 study focused on the association between early rhythm control and cardiovascular outcomes [17]. Consistent with the findings from our analysis, the study reported a lower risk of death from cardiovascular causes, and a lower occurrence of the composite of Death/Stroke/aHF/MI in the early rhythm control group. Whereas only approximately one fifth of patients in the early rhythm control group received an AF ablation after 2 years, the authors described a significant impact on the superiority of the early rhythm control group as conceivable. Whereas our main research question limits the comparison to a PVI and non-PVI group, we also performed a survival analysis comparing patients receiving rhythm control (PVI or antiarrhythmic drugs) and patients without rhythm control (consistent with the research question of the EAST-AFNET 4 trial). We found a mitigated effected compared to our main analysis with regard to mortality, but a consistent effect regarding the composite endpoint of Death/Stroke/aHF/MI, as shown in Fig. S6 in the supplement.

The EAST-AFNET 4 trial reported no significant benefit of early rhythm control regarding hospitalizations with worsening heart failure despite including a similar proportion of heart failure patients compared to our cohorts [17]. However, in EAST-AFNET 4, there were also numerically fewer heart failure hospitalizations in the early rhythm control group with a hazard ratio of 0.81 (95%CI 0.65–1.02) [17]. That said, a recent subanalysis assessing the effect of early rhythm control compared to usual care in patients with heart failure demonstrated a significant reduction in the composite of all-cause death or hospitalization for worsening of heart failure [21].

In CASTLE-AF [13], a trial including heart failure patients, the reported rate of hospitalizations for worsening heart failure was lower in the ablation group, which supports our findings in a real world AF population with lower heart failure prevalence. For a broader comparison, we conducted the main analysis with an additional endpoint modelled after CASTLE-AF (composite of all-cause death or hospital admission for acute heart failure) and included this in Fig. S7 and Table S4 in the supplement. The lower incidence of hospital admissions in the PVI group in our population could be a result of the favourable effects of rhythm control, but residual confounding between the PVI and non-PVI group is likely. While we aimed to reduce the differences in comorbidities by matching, we still observed a higher burden of coronary artery disease and heart failure in the non-PVI group. This may contribute to the higher rate of hospital admissions in the non-PVI group. Despite the higher burden of coronary artery disease in the non-PVI group, we did not observe a significantly higher rate of MI. We conducted a sensitivity analysis without defining a matched population showing consistent results compared to our main analysis.

In our study, the incidence of stroke/TIA/SE was low both in the PVI and non-PVI groups, with no significant difference between the two groups. These findings differ from those of a previous large observational study where patients who did not undergo ablation where matched to an AF ablation group and to a population with no history of AF [22]. The authors reported a significantly lower risk of stroke in AF ablation patients compared to AF patients who did not undergo ablation. However, their matching was just based on age and sex, which resulted in an uneven distribution of variables such as heart failure, valvular heart disease and hypertension across the different groups. The EAST-AFNET 4 trial reported a significant reduction of stroke risk in the early rhythm control group, although event rates were low in both groups [17]. However, with a CHA_2_DS_2_-VASc score of 3.4 in the early rhythm control group, EAST-AFNET 4 included higher risk patients compared to our study (mean CHA_2_DS_2_-VASc score of 2.4). Furthermore, they included only patients with early AF (diagnosed ≤ 1 year before enrolment), whereas in our study the median time between AF diagnosis and study enrolment was 3.5 years. Knowing that the risk of cardiovascular complications is increased during the first year after AF diagnosis [23], it could be hypothesized that the effect on the risk reduction is more pronounced in patients with early AF. However, our results support the ongoing use of oral anticoagulation in AF patients with an increased risk of stroke, independent on whether or not patients have undergone PVI.

Finally, the performed sensitivity analysis in the unmatched, time-updated population provided consistent results compared to our main analysis in the matched population. In both populations, the PVI group reported a significantly lower rate of all-cause mortality and hospital admissions for acute heart failure compared to the non-PVI group, although the association was weaker in the unmatched, time-updated population.

### Study strengths and limitations

The main strength of our analysis is the well-characterized AF patient population with a median follow-up time of 3.9 years. Additionally, the number of missing values was very low and clinical information was obtained on a yearly basis during follow-up. On the other hand, several limitations should be noted. First, we only included a matched selection of our study population in the analyses and controlled for important potential confounders. Yet, some unaccounted confounders likely persist, and this analysis therefore cannot establish causality. Second, by excluding patients who did not survive to the first follow-up visit, we may have introduced a survival bias. However, the sensitivity analysis which included this time period resulted in similar findings making this hypothesis unlikely. Third, the power to detect differences in rare outcomes (e.g. stroke, acute coronary syndrome) is rather low. Finally, this study compared two strategies, not the success of PVI in terms of symptomatic arrhythmia recurrence after PVI, because rhythm outcome information was not systematically available for all patients. This study focused on major cardiovascular events and death, not on rhythm-related outcomes.

## Conclusions

In a matched comparison, AF patients undergoing PVI had a lower risk of all-cause mortality and hospital admission for acute heart failure, but not for stroke/TIA/SE, MI, or bleeding compared to patients who did not undergo PVI.

## Supplementary Information

Below is the link to the electronic supplementary material.

Supplementary file1 (DOCX 759 KB)
