# Supplementary material for: Association of pulmonary vein isolation and major cardiovascular events in patients with atrial fibrillation
Source: Clin Res Cardiol. 2022 Apr 11;111(9):1048–56. doi: 10.1007/s00392-022-02015-0 (PMC9424150; doi:10.1007/s00392-022-02015-0)
Supplement: Supplementary file 1 — Supplementary file1 (DOCX 759 KB) [file 392_2022_2015_MOESM1_ESM.docx]

**Association of Pulmonary Vein Isolation and cardiovascular outcome events in patients with atrial fibrillation**

Marc Girod^1,2^, Michael Coslovsky^1,2,3^, Stefanie Aeschbacher^1,2^, Christian Sticherling^1,2^, Tobias Reichlin^4^, Laurent Roten^4^, Nicolas Rodondi^5,6^, Peter Ammann^7^, Angelo Auricchio^8^, Giorgio Moschovitis^9^, Richard Kobza^10^, Patrick Badertscher^1,2^, Sven Knecht^1,2^, Philipp Krisai^1,2^, Andrea Marugg^1,2^, Helena Aebersold^11^, Elisa Hennings^1,2^, Miquel Serra-Burriel^11^, Matthias Schwenkglenks^11,12^, Christine S. Zuern^1,2^, Leo H Bonati^13^, David Conen^14^, Stefan Osswald^1,2^, Michael Kühne^1,2^

1. Cardiovascular Research Institute Basel, University Hospital Basel, University of Basel, Switzerland
2. Department of Cardiology, Department of Medicine, University Hospital Basel, University of Basel, Switzerland
3. Clinical Trial Unit Basel, Department of Clinical Research, University Hospital Basel, Switzerland
4. Department of Cardiology, Inselspital, Bern University Hospital, University of Bern, Bern, Switzerland.
5. Institute of Primary Health Care (BIHAM), University of Bern, Switzerland
6. Department of General Medicine, Inselspital, Bern University Hospital, University of Bern, Switzerland
7. Department of Cardiology, Kantonsspital St. Gallen, St. Gallen Switzerland
8. Division of Cardiology, Institute Cardiocentro Ticino, Lugano, Switzerland
9. Division of Cardiology, Ente Ospedaliero Cantonale, Regional Hospital of Lugano, Lugano, Switzerland
10. Department of Cardiology, Luzerner Kantonsspital, Switzerland
11. Epidemiology, Biostatistics, and Prevention Institute, University of Zürich, Zürich, Switzerland
12. Institute of Pharmaceutical Medicine, University of Basel, Basel, Switzerland
13. Department of Neurology and Stroke Center, University Hospital Basel, University of Basel, Basel, Switzerland
14. Population Health Research Institute, McMaster University, Hamilton, Canada

| Supplementary Table S1. | Definition of study outcome events |
| --- | --- |
| Supplementary Table S2. | Sensitivity analysis – Baseline characteristics of a time-updated study population |
| Supplementary Table S3. | Sensitivity analysis – Multivariable adjusted cox-proportional hazards models in a time-updated study population |
| Supplementary Table S4 | Supplementary Table S4. Multivariable adjusted cox-proportional hazards models in a matched population – including additional composite |
| Supplementary Figure S1. | Study population of the matched population using Coarsened Exact Matching (CEM) |
| Supplementary Figure S2. | Standardized mean difference (SMD) between the two groups after matching |
| Supplementary Figure S3.1-S3.5. | Cumulative incidence curves for each outcome event in a matched population |
| Supplementary Figure S4. | Study population of the time-updated sensitivity analysis |
| Supplementary Figure S5. | Multivariable adjusted cox-proportional hazards models – Matched vs time-updated population |
| Supplementary Figure S6. | Multivariable adjusted cox-proportional hazards models for adverse events in a matched population, comparing a rhythm control group (PVI or antiarrhythmic drugs) to a no rhythm control group. |
| Supplementary Figure S7. | Multivariable adjusted cox-proportional hazards models for adverse events in a matched population – including a composite of all-cause death and heart failure hospitalisation |

**Supplementary Table S1.** Definition of study outcome events

| All-cause mortality |
| --- |
| Deaths were classified as cardiovascular or non-cardiovascular origin. All deaths were assumed to be of cardiovascular origin unless a non-cardiovascular cause could be clearly established. |
| Hospital admission for acute heart failure |
| Hospitalization for acute heart failure is defined as: any hospitalization for acute heart failure that is associated with at least one overnight stay. If it is not clear whether the reason for a patient’s hospitalization is acute heart failure or not, this event/incidence should in doubt be classified as acute heart failure.  The following references mentioned under clinical examination, or in the progress entry could be used as an indication for heart failure: leg swelling/leg oedema, distension of the neck veins, positive hepato-jugular reflux, rales, and 3rd heart sound. |
| Stroke/TIA and systemic embolism |
| Stroke was defined as an acute focal neurologic deficit of vascular origin, confirmed by imaging or autopsy. Stroke was categorized as ischemic, haemorrhagic, or undetermined.   - Ischemic stroke: rapid onset of focal neurological dysfunction with clinical, imaging, or pathological evidence explaining the dysfunction. Clinical evidence of infarction is based on symptoms persisting ≥24 hours or until death, and exclusion of other aetiologies. - Haemorrhagic stroke: rapid onset of focal or global neurological dysfunction and/or headache attributable to a focal collection of blood within the brain parenchyma or ventricular system that is not caused by trauma. - If the type of stroke cannot be determined by imaging or other means (e.g., lumbar puncture, neurosurgery, or autopsy) but is judged to fulfil the stroke definition above, the stroke will be classified as undetermined stroke.   Transient ischemic attack (TIA) was defined as a transient episode of neurologic dysfunction caused by focal brain, spinal cord, or retinal ischemia without cerebral infarction on imaging.  Systemic arterial embolism was considered to have occurred if there were clinical signs and symptoms consistent with embolic arterial occlusion. And at least one of the following objective findings: Surgical report indicating evidence of arterial embolism, pathological specimens related to embolism removal, imaging evidence consistent with arterial embolism and/or autopsy reports. |
| Myocardial infarction |
| Myocardial infarction was defined according to the universal definition of MI as rise and/or fall of cardiac troponin with at least one value above the 99th percentile of the upper reference limit in a clinical setting consistent with myocardial ischemia, and with at least one of the following: symptoms of ischemia, new ST elevation and/or new horizontal or down-sloping ST depression in two contiguous leads on ECG; new left bundle brunch block on ECG; development of pathological Q waves on ECG; imaging evidence of new loss of viable myocardium or new regional wall motion; identification of an intracoronary thrombus by angiography or autopsy. |
| Major bleeding and clinically relevant non-major bleeding |
| Bleeding was defined according to the International Society on Thrombosis and Haemostasis criteria [1].  Major bleeding was defined as clinically overt bleeding with a fatal outcome or a reduction in haemoglobin level of ≥20g/l within 7 days, or transfusion of at least two units of blood, or symptomatic bleeding in a critical area or organ (intracranial, intraspinal, intraocular, pericardial, intra-articular, intramuscular with compartment syndrome, retroperitoneal).  Clinically relevant non-major bleeding was defined as an overt bleeding event, which satisfies none of the additional criteria mentioned above and that leads to either hospital admission, or physician guided medical or surgical treatment, or a change in antithrombotic therapy. |

**Supplementary Table S2.** Sensitivity analysis – Baseline characteristics of a time-updated study population

| Characteristic | Overall (n=3885) |
| --- | --- |
| Age, y (median [IQR]) | 72.4 [66.1, 78.1] |
| Female sex, n (%) | 1092 (28.1) |
| BMI, kg/m^2^ (median [IQR]) | 26.7 [24.2 30.0] |
| Active smoker, n (%) | 304 (7.8) |
| Education level |  |
| Basic | 462 (12.0) |
| Middle | 1903 (49.2) |
| Advanced | 1499 (38.8) |
| AF type, n (%) |  |
| Paroxysmal | 1910 (49.2) |
| Non-paroxysmal | 1975 (50.8) |
| Time since AF diagnosis, y (median [IQR]) | 3.2 [0.9, 7.6] |
| Medical history |  |
| Stroke or TIA, n (%) | 664 (17.1) |
| Hypertension, n (%) | 2675 (68.9) |
| Heart failure, n (%) | 925 (23.8) |
| Coronary artery disease n (%) | 1041 (26.8) |
| Myocardial infarction, n (%) | 573 (14.7) |
| Diabetes, n (%) | 619 (15.9) |
| Renal failure, n (%) | 722 (18.6) |
| RFA of atrial flutter, n (%) | 466 (12.0) |
| ECV, n (%) | 1342 (34.6) |
| PTCA, n (%) | 778 (20.0) |
| CABG, n (%) | 364 (9.4) |
| PVI, n (%) | 824 (21.2) |
| Medication |  |
| Oral anticoagulation, n (%) | 3271 (84.2) |
| Antiplatelet therapy, n (%) | 836 (21.6) |
| Antiarrhythmic drugs, n (%) | 907 (23.3) |
| Betablocker, n (%) | 2676 (68.9) |
| CHA_2_DS_2_-VASc score (mean, SD) | 3.2 (1.7) |
| Values presented as mean ± SD, median (interquartile range) or n (%).  CHA2DS2-VASc = congestive heart failure, hypertension, age ≥75 yrs. (2 points), diabetes, prior stroke or TIA or thromboembolism (2 points), vascular disease, age 65 to 74 yrs., female sex. IQR = interquartile range; BMI = body mass index; AF = atrial fibrillation; TIA = transient ischemic attack; RFA = radiofrequency ablation; ECV = electrical cardioversion; PTCA = percutaneous transluminal coronary angioplasty; CABG = coronary artery bypass graft  Missing values: BMI (n=5), Smoking (n=10), Education level (n=21), Time since AF diagnosis (n=532), Stroke/TIA (n=1), Oral anticoagulation (n=1), Antiplatelet therapy (n=17), RFA isthmus (n=4), ECV (n=3), CHA2DS2-VASc score (n=1)  Patients with history of PVI at baseline were not excluded for further analysis. | |

**Supplementary Table S3.** Sensitivity analysis – Multivariable adjusted cox-proportional hazards models in a time-updated study population

|  | Group | Number of events | Incidence rate  (per 100 person years) | Model 1* | | Model 2 † | |
| --- | --- | --- | --- | --- | --- | --- | --- |
|  |  |  |  | **Hazard Ratio**  **(95% CI)** | **p-value** | **Hazard Ratio**  **(95% CI)** | **p-value** |
| Adverse outcome events | | | | | | | |
| All-cause mortality | PVI | 13 | 0.54 | 0.31 (0.18, 0.55) | <0.001 | 0.40 (0.23, 0.71) | 0.002 |
|  | Non-PVI | 549 | 3.46 |  |  |  |  |
| Hospital admission for acute heart failure | PVI | 19 | 0.81 | 0.45 (0.28, 0.72) | <0.001 | 0.57 (0.35, 0.91) | 0.019 |
|  | Non-PVI | 531 | 3.56 |  |  |  |  |
| Stroke/TIA and systemic embolism | PVI | 10 | 0.42 | 0.59 (0.30, 1.14) | 0.11 | 0.62 (0.32, 1.21) | 0.2 |
|  | Non-PVI | 177 | 1.14 |  |  |  |  |
| Myocardial infarction | PVI | 4 | 0.17 | 0.28 (0.10, 0.78) | 0.015 | 0.36 (0.13, 1.00) | 0.049 |
|  | Non-PVI | 127 | 0.81 |  |  |  |  |
| Major and clinically relevant non-major bleeding | PVI | 48 | 2.15 | 0.73 (0.54, 0.99) | 0.044 | 0.79 (0.58, 1.07) | 0.12 |
|  | Non-PVI | 685 | 4.78 |  |  |  |  |
| PVI status, outcome events and covariates used in the models were time-updated.  Model 1 was adjusted for age and sex. Model 2 was additionally adjusted for AF type, history of hypertension, diabetes, coronary artery disease and heart failure hospitalizations. Patients with history of PVI at baseline were not excluded for further analysis. | | | | | | | |

**Supplementary Table S4.** Multivariable adjusted cox-proportional hazards models in a matched population – including additional composite

|  | Group* | Number of events | Incidence rate  (per 100 person years) | Model 1† | | Model 2‡ | |
| --- | --- | --- | --- | --- | --- | --- | --- |
|  |  |  |  | **Hazard Ratio**  **(95% CI)** | **p-value** | **Hazard Ratio**  **(95% CI)** | **p-value** |
| Adverse outcome events | | | | | | | |
| All-cause mortality | PVI | 10 | 0.64 | 0.35 (0.18, 0.70) | 0.003 | 0.39 (0.19, 0.79) | 0.009 |
|  | Non-PVI | 163 | 1.87 |  |  |  |  |
| Hospital admission for acute heart failure | PVI | 8 | 0.52 | 0.34 (0.16, 0.70) | 0.003 | 0.44 (0.21, 0.95) | 0.035 |
|  | Non-PVI | 146 | 1.72 |  |  |  |  |
| Stroke/TIA and systemic embolism | PVI | 14 | 0.91 | 0.89 (0.49, 1.60) | 0.7 | 0.94 (0.52, 1.69) | 0.8 |
|  | Non-PVI | 93 | 1.09 |  |  |  |  |
| Myocardial infarction | PVI | 3 | 0.19 | 0.28 (0.08, 0.95) | 0.041 | 0.43 (0.11, 1.63) | 0.2 |
|  | Non-PVI | 50 | 0.58 |  |  |  |  |
| Major and clinically relevant non-major bleeding | PVI | 32 | 2.16 | 0.70 (0.47, 1.04) | 0.078 | 0.75 (0.50, 1.12) | 0.2 |
|  | Non-PVI | 266 | 3.27 |  |  |  |  |
| Composite of death from cardiovascular causes, stroke, or hospital admission for acute heart failure or myocardial infarction | PVI | 26 | 1.72 | 0.52 (0.34, 0.79) | 0.002 | 0.63 (0.40, 0.97) | 0.038 |
|  | Non-PVI | 306 | 3.46 |  |  |  |  |
| Composite of all-cause death or hospital admission for acute heart failure | PVI | 16 | 1.04 | 0.40 (0.24, 0.68) | <0.001 | 0.48 (0.28, 0.83) | 0.008 |
|  | Non-PVI | 256 | 2.83 |  |  |  |  |
| * Matching was based on age categories, sex, AF type, history of diabetes and history of hypertension using Coarsened Exact Matching (CEM). To account for the effect of different strata sizes, weights generated throughout the matching process were applied. Patients with a history of PVI at baseline were not eligible for the control group. † Model 1 was adjusted for age, within each stratum. ‡ Model 2 was additionally adjusted for history of coronary artery disease and heart failure, within each stratum. | | | | | | | |

**3’968** patients enroled in BEAT-AF and Swiss-AF

**3’607** patients stratified by PVI status in a one-year observational period

PVI group (n=325)

Non-PVI group (n=3’282)

**Exclusion of patients with:**

Accidental enrolment in both studies (n=7)

Missing information on PVI status (n=313)

Study termination without follow-up information (n=41)

**2’934** patients eligible for matching:

PVI group (n=325)

Non-PVI group (n=2’609)

**2’518** patients matched using Coarsened Exact Matching

Non-PVI group (**n=2193**)

PVI group (**n=325**)

**416** patients could not be matched

**Exclusion of patients in non-PVI population with:**

History of PVI before study enrollment (n=673)

**Supplementary Figure S1.** Study population of the matched population using Coarsened Exact Matching (CEM)

***
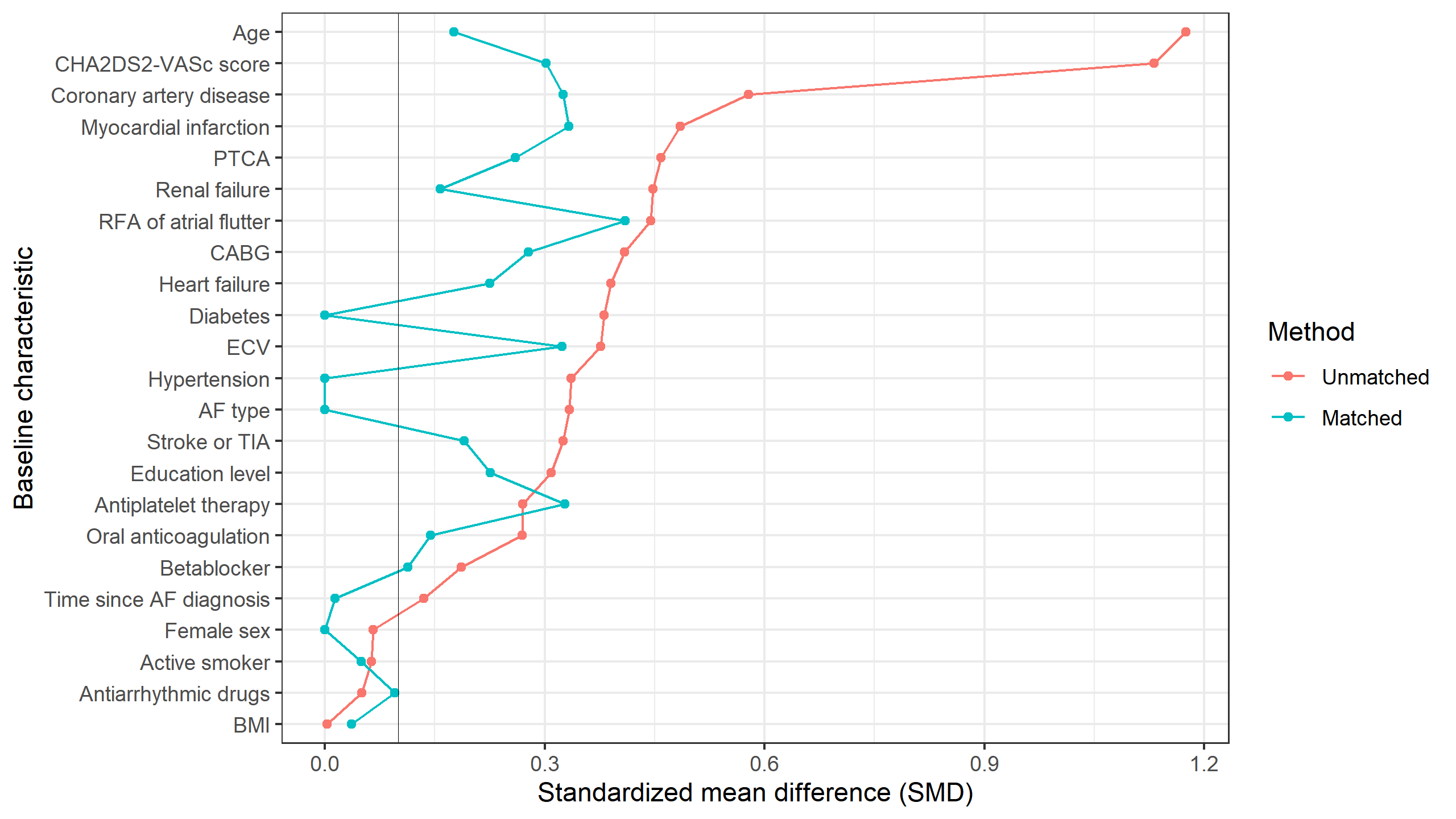
***

**Supplementary Figure S2.** Standardized mean difference (SMD) between the two groups after matching

CHA2DS2-VASc = congestive heart failure, hypertension, age ≥75 yrs. (2 points), diabetes, prior stroke or TIA or thromboembolism (2 points), vascular disease, age 65 to 74 yrs., female sex. SMD = standardized mean difference; BMI = body mass index; AF = atrial fibrillation; TIA = transient ischemic attack; RFA = radiofrequency ablation; ECV = electrical cardioversion; PTCA = percutaneous transluminal coronary angioplasty; CABG = coronary artery bypass graft

***
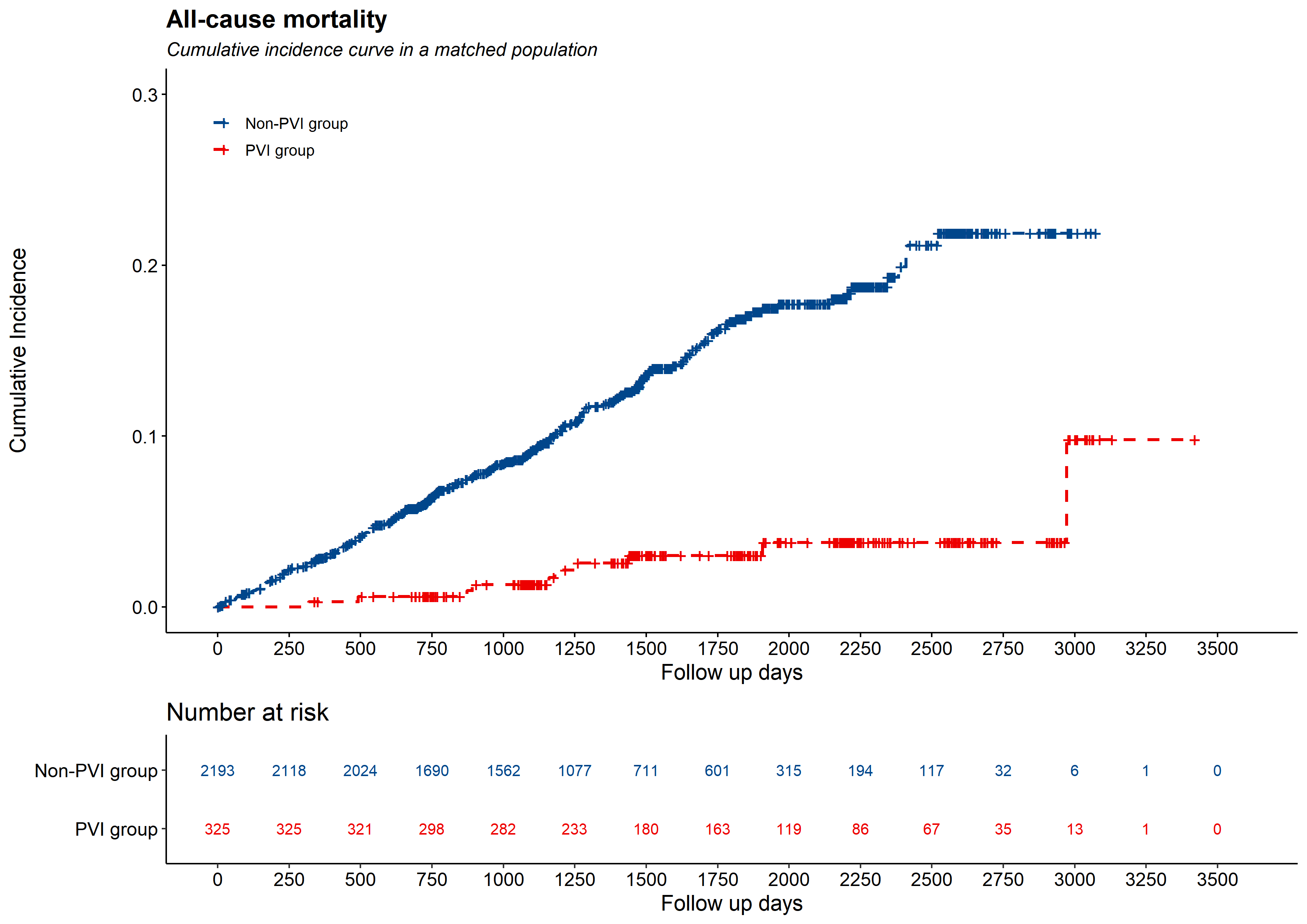
***

**Supplementary Figure S3.1.** All-cause mortality – Cumulative incidence curve in a matched population

***
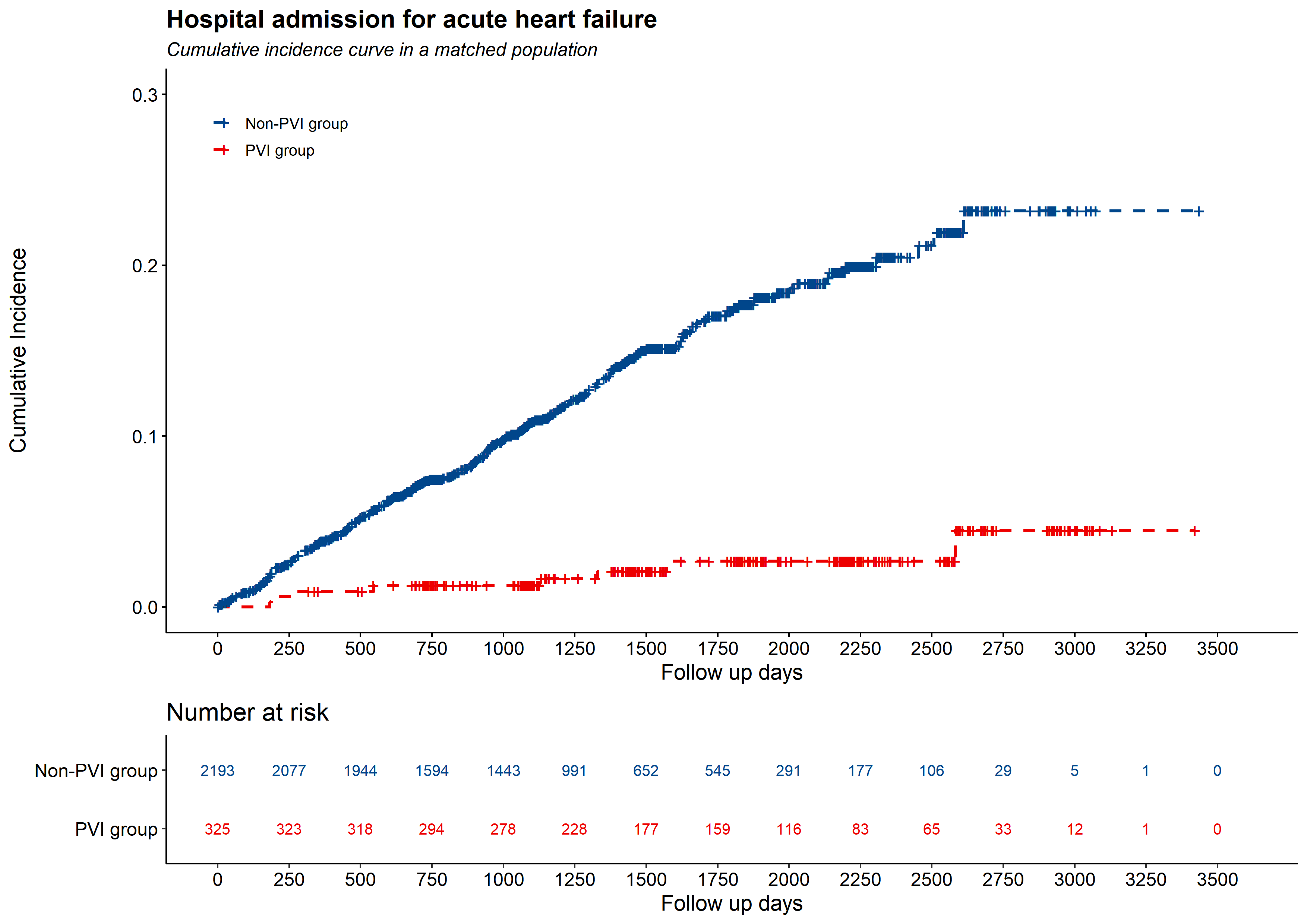
***

**Supplementary Figure S3.2.** Hospital admission for acute heart failure – Cumulative incidence curve in a matched population

***
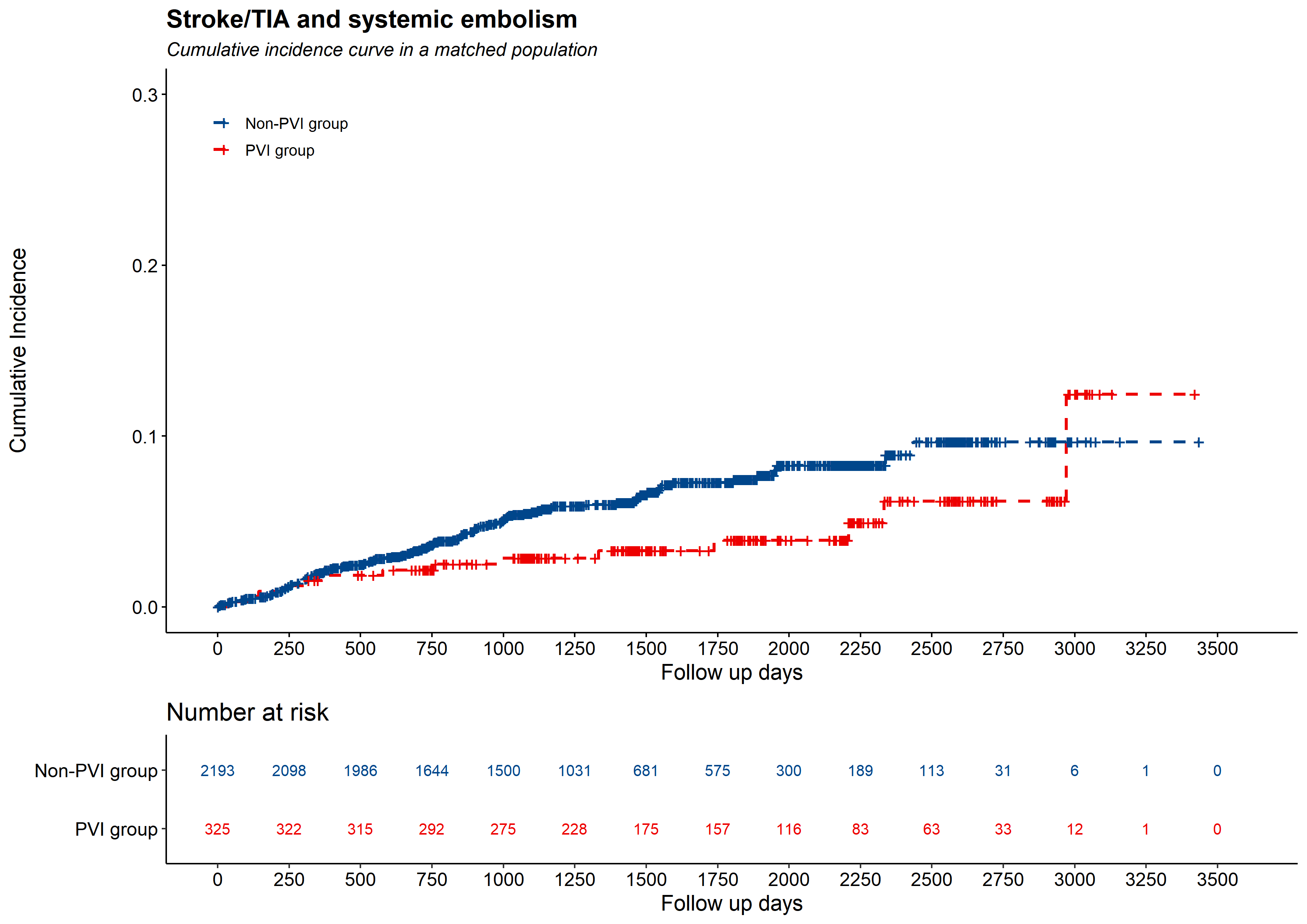
***

**Supplementary Figure S3.3.** Stroke/TIA and systemic embolism – Cumulative incidence curve in a matched population

***
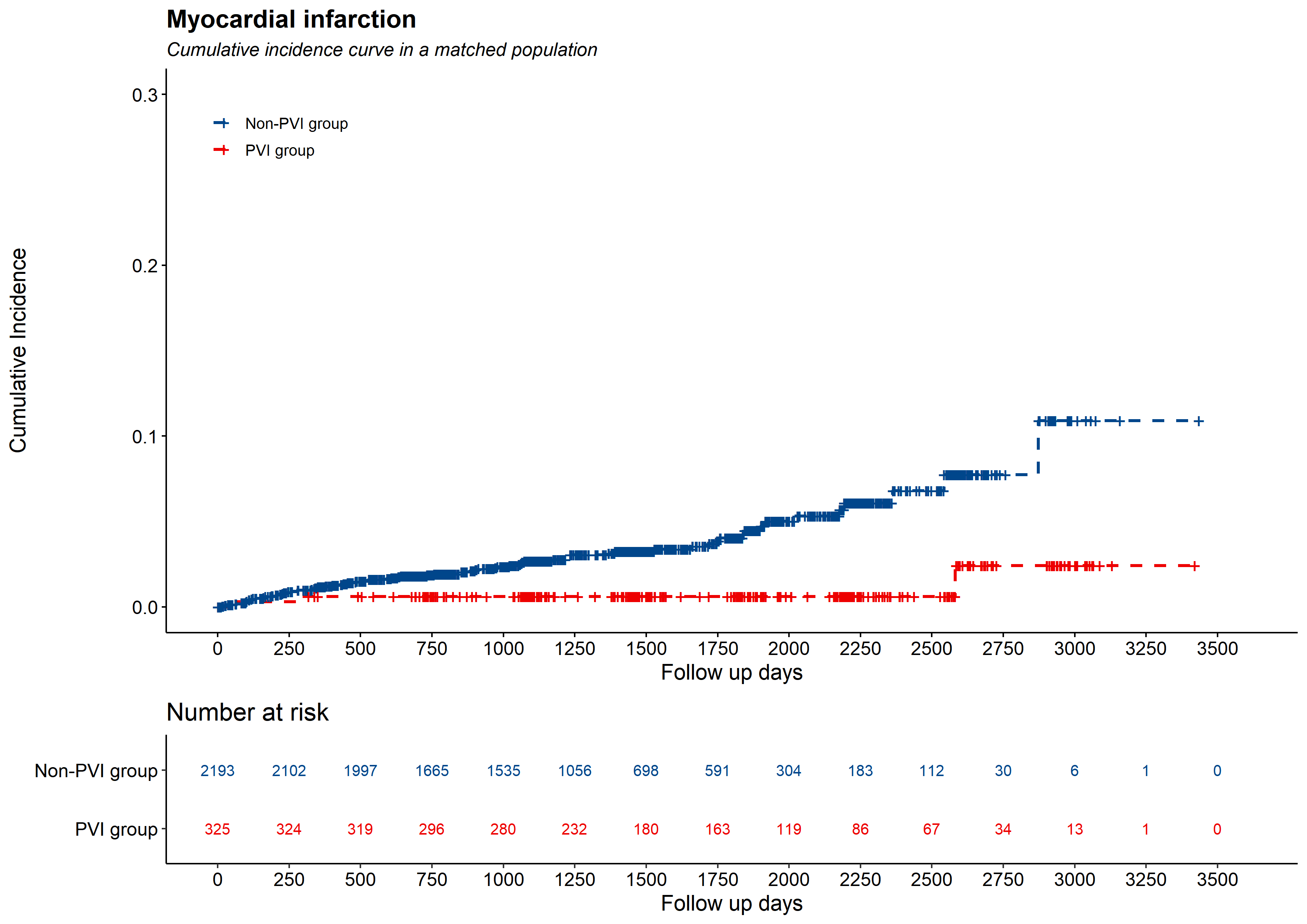
***

**Supplementary Figure S3.4.** Myocardial infarction – Cumulative incidence curve in a matched population

***
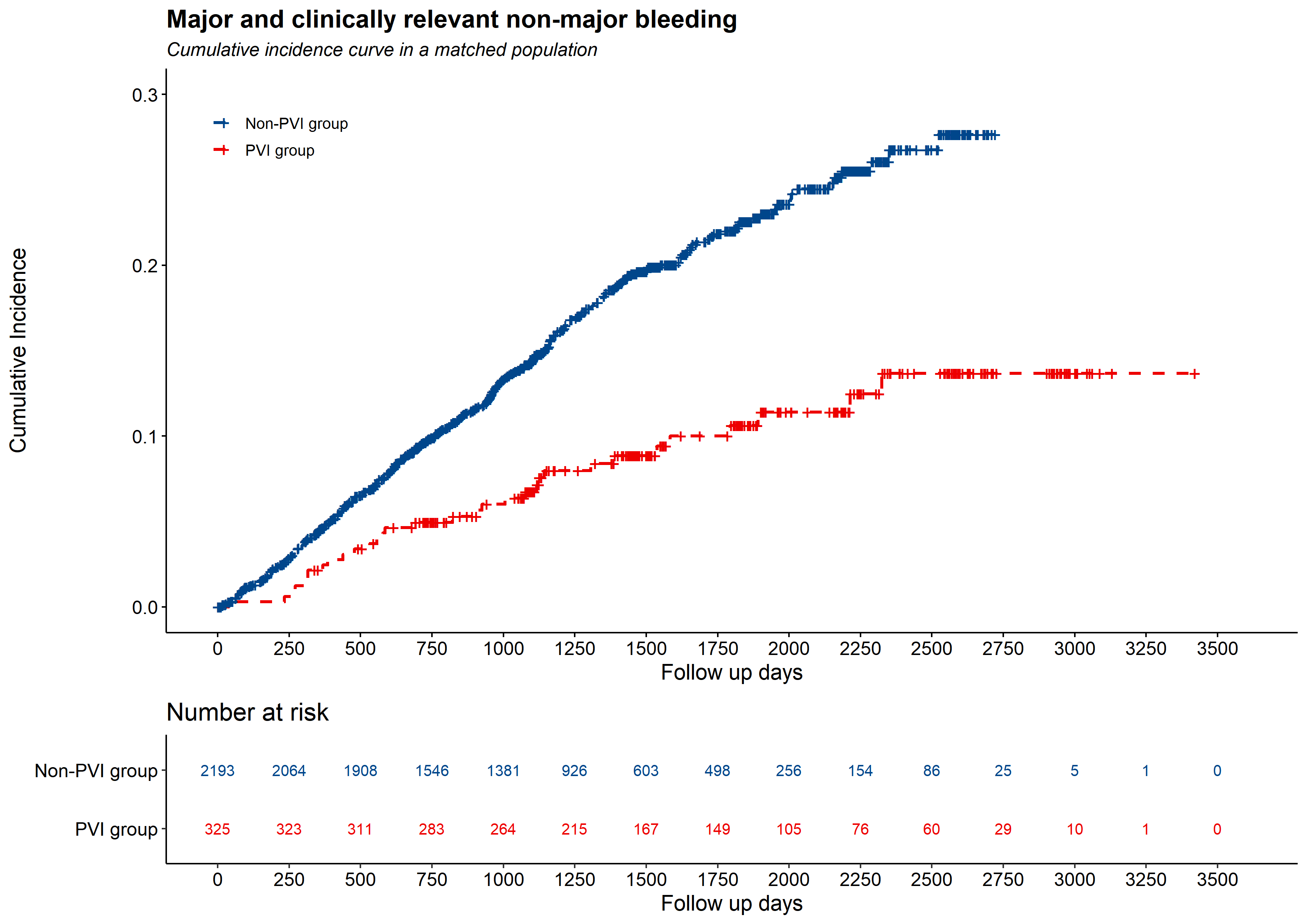
***

**Supplementary Figure S3.5.** Major and clinically relevant non-major bleeding – Cumulative incidence curve in a matched population


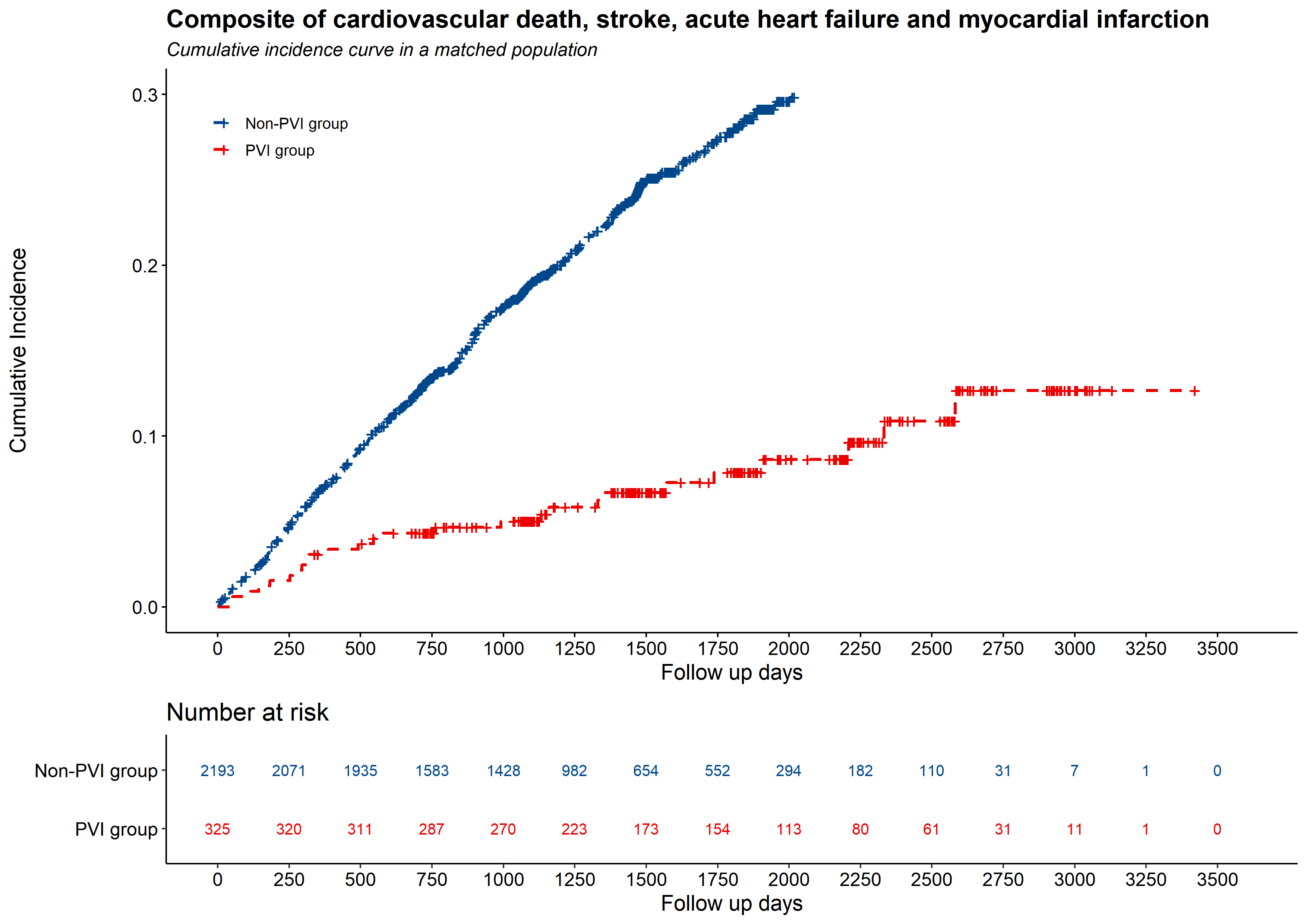


**Supplementary Figure S3.6.** Composite endpoint consisting of death from cardiovascular causes, stroke, or hospital admission for acute heart failure or myocardial infarction – Cumulative incidence curve in a matched population

**3’968** patients enroled in BEAT-AF and Swiss-AF

**3’952** patients before time-update of covariates

**Exclusion of patients with:**

Accidental enrolment in both studies (n=7)

Missing information on important covariates at baseline (n=9)

**3’885** patients eligible for the final analysis

**Exclusion of patients with:**

Study termination without any follow-up information (n=67)

**Supplementary Figure S4.** Study population of the time-updated sensitivity analysis


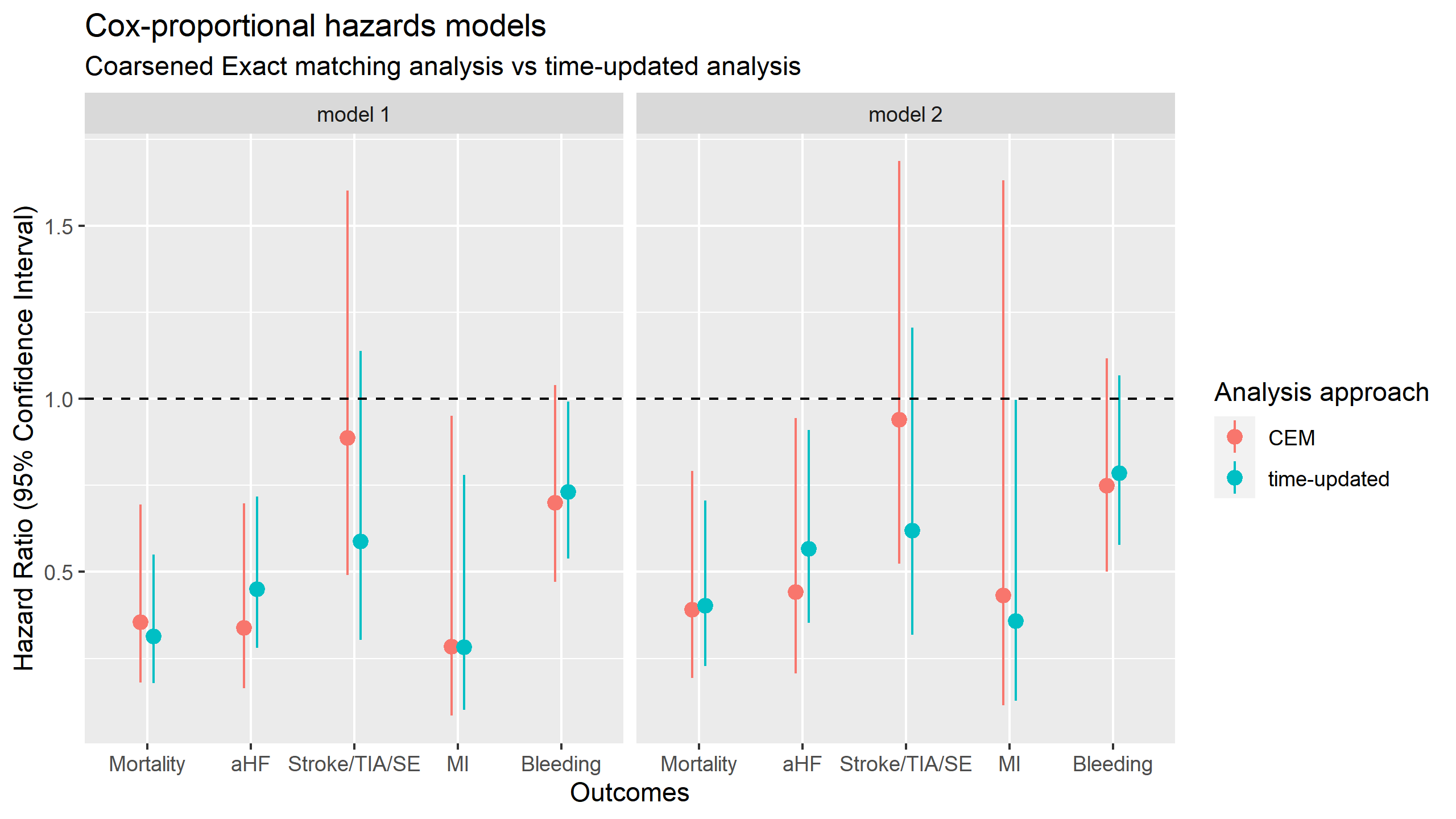


**Supplementary Figure S5.** Multivariable adjusted cox-proportional hazards models – Matched (CEM) vs time-updated population

Mortality = All-cause mortality; aHF = Hospital admission for acute heart failure; Stroke/TIA/SE = Composite of stroke, transient ischemic attack, and systemic embolism; MI = Myocardial infarction; Bleeding = Composite of major and clinically relevant non-major bleeding

Time-updated analysis: PVI status, outcome events and covariates used in the models were time-updated. Model 1 was adjusted for age and sex. Model 2 was additionally adjusted for AF type, history of hypertension, diabetes, coronary artery disease and heart failure hospitalizations. Patients with history of PVI at baseline were not excluded for further analysis.

Coarsened Exact Matching analysis (CEM): Model 1 was adjusted for age, within each stratum. Model 2 was additionally adjusted for history of coronary artery disease and heart failure hospitalizations. Patients with a history of PVI at baseline were excluded of the control group


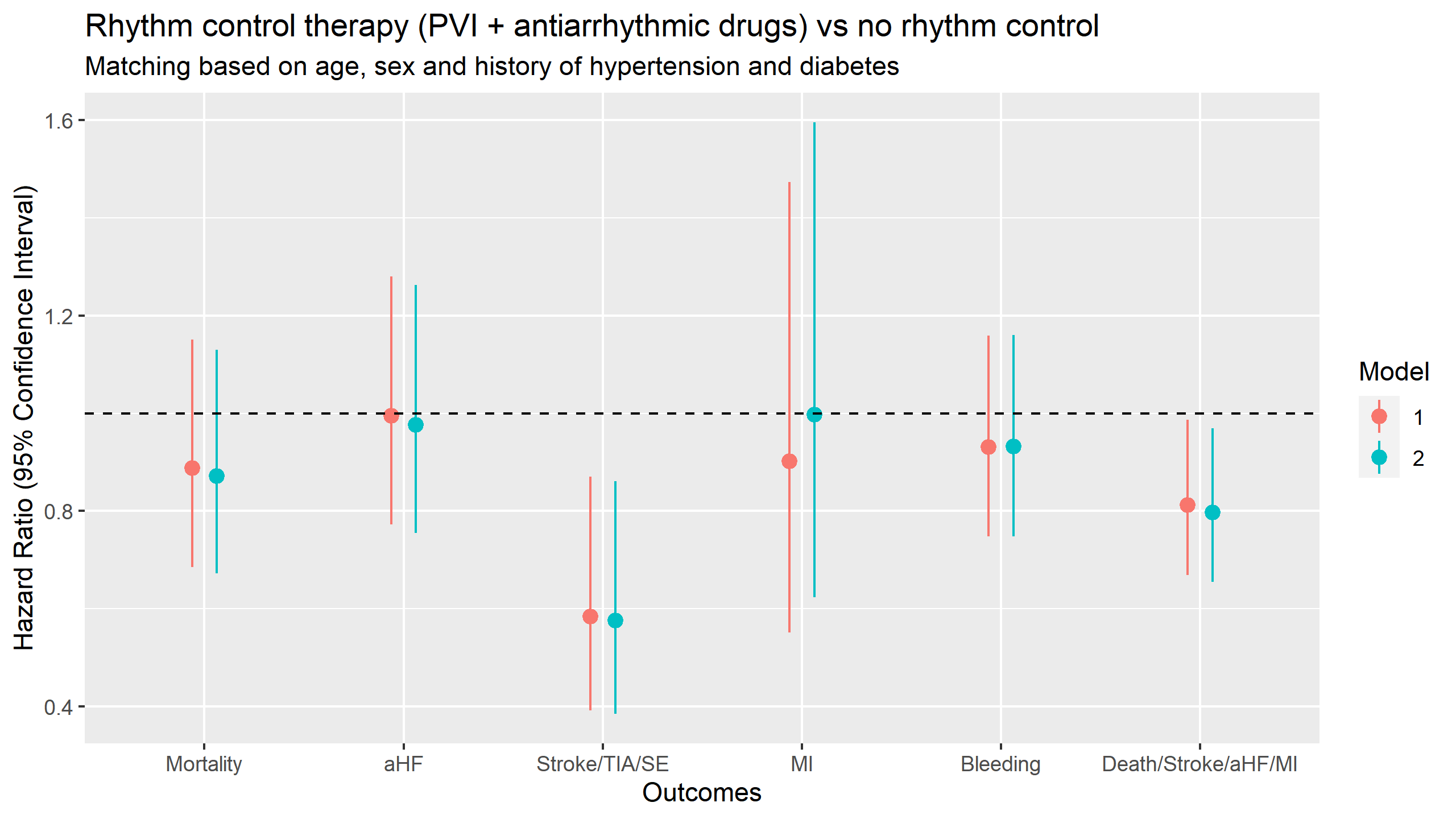


**Supplementary Figure S6.** Multivariable adjusted cox-proportional hazards models for adverse events in a matched population, comparing patients receiving rhythm control (PVI or antiarrhythmic drugs) to patients without rhythm control.

a) All-cause mortality (Mortality); b) Hospital admission for acute heart failure (aHF); c) Stroke, transient ischemic attack, and systemic embolism (Stroke/TIA/SE); d) Myocardial infarction (MI); e) Major bleeding and clinically relevant non-major bleeding (Bleeding), f) composite of death from cardiovascular causes, stroke, or hospital admission for acute heart failure or myocardial infarction (Death/Stroke/aHF/MI). The colours represent the two different models. Model 1 was adjusted for age, within each stratum with identical values for all matching covariates. Model 2 was additionally adjusted for history of coronary artery disease and heart failure hospitalization, within each stratum with identical values for all matching covariates.


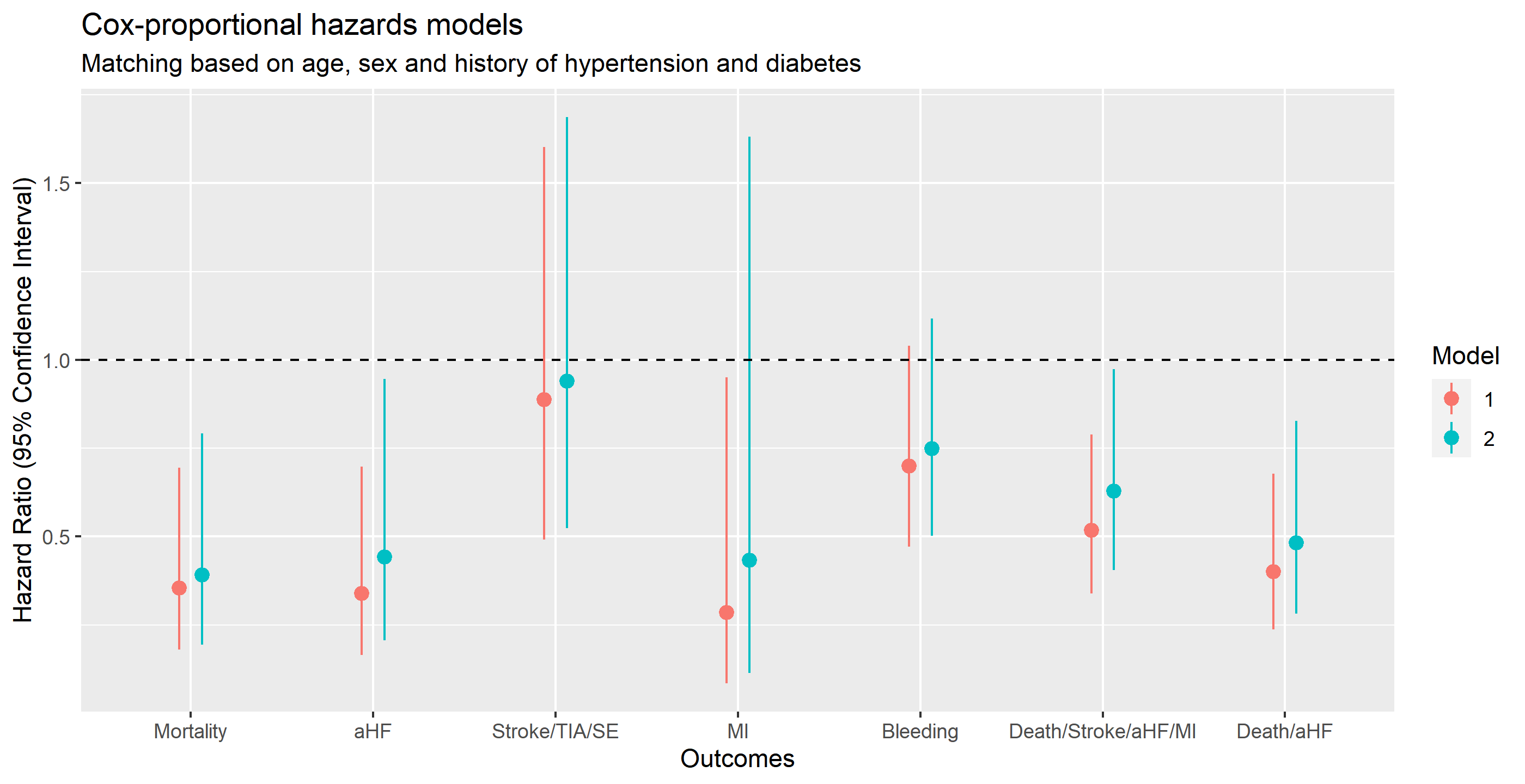


**Supplementary Figure S7.** Multivariable adjusted cox-proportional hazards models for adverse events in a matched population – comparison between a PVI and non-PVI group

a) All-cause mortality (Mortality); b) Hospital admission for acute heart failure (aHF); c) Stroke, transient ischemic attack, and systemic embolism (Stroke/TIA/SE); d) Myocardial infarction (MI); e) Major bleeding and clinically relevant non-major bleeding (Bleeding), f) Composite of death from cardiovascular causes, stroke, or hospital admission for acute heart failure or myocardial infarction (Death/Stroke/aHF/MI), g) Composite of all-cause death or hospital admission for acute heart failure. The colours represent the two different models. Model 1 was adjusted for age, within each stratum with identical values for all matching covariates. Model 2 was additionally adjusted for history of coronary artery disease and heart failure hospitalization, within each stratum with identical values for all matching covariates.

**Swiss-AF and BEAT-AF investigators**

University Hospital Basel and Basel University: Stefanie Aeschbacher, Chloé Auberson, Steffen Blum, Leo Bonati, Selinda Ceylan, David Conen, Simone Evers- Doerpfeld, Ceylan Eken, Marc Girod, Elisa Hennings, Elena Herber, Vasco Iten, Philipp Krisai, Michael Kühne, Mirko Lischer, Christine Meyer-Zürn, Pascal Meyre, Andreas U. Monsch, Christian Müller, Stefan Osswald, Anne Springer, Christian Sticherling, Thomas Szucs, Gian Völlmin.

Principal Investigator: Stefan Osswald; Local Principal Investigator: Michael Kühne

University Hospital Bern: Faculty: Drahomir Aujesky, Urs Fischer, Juerg Fuhrer, Laurent Roten, Simon Jung, Heinrich Mattle; Research fellows: Luise Adam, Carole Elodie Aubert, Martin Feller, Axel Loewe, Elisavet Moutzouri, Claudio Schneider; Study nurses: Tanja Flückiger, Cindy Groen, Lukas Ehrsam, Sven Hellrigl, Alexandra Nuoffer, Damiana Rakovic, Nathalie Schwab, Rylana Wenger. Local Principal Investigator: Nicolas Rodondi

Stadtspital Triemli Zurich: Christopher Beynon, Roger Dillier, Michèle Deubelbeiss,

Franz Eberli, Christine Franzini, Isabel Juchli, Claudia Liedtke, Jacqueline Nadler, Thayze Obst, Jasmin Roth, Fiona Schlomowitsch, Xiaoye Schneider, Katrin Studerus, Noreen Tynan, Dominik Weishaupt. Local Principal Investigator: Andreas Müller

Kantonspital Baden: Simone Fontana, Silke Kuest, Karin Scheuch, Denise Hischier, Nicole Bonetti, Alexandra Grau, Jonas Villinger, Eva Laube, Philipp Baumgartner, Mark Filipovic, Marcel Frick, Giulia Montrasio, Stefanie Leuenberger, Franziska Rutz. Local Principal Investigator: Jürg-Hans Beer

Cardiocentro Lugano: Angelo Auricchio, Adriana Anesini, Cristina Camporini, Giulio Conte, Maria Luce Caputo, Francois Regoli. Local Principal Investigator: Tiziano Moccetti

Kantonsspital St. Gallen: Roman Brenner, David Altmann, Michaela Gemperle. Local Principal Investigator: Peter Ammann

Hôpital Cantonal Fribourg: Mathieu Firmann, Sandrine Foucras, Martine Rime. Local Principal Investigator: Daniel Hayoz

Luzerner Kantonsspital: Benjamin Berte, Virgina Justi, Frauke Kellner-Weldon, Brigitta Mehmann, Sonja Meier, Myriam Roth, Andrea Ruckli-Kaeppeli, Ian Russi, Kai Schmidt, Mabelle Young, Melanie Zbinden. Local Principal Investigator: Richard Kobza

Ente Ospedaliero Cantonale Lugano: Maria Luisa De Perna, Jane Frangi-Kultalahti, Anica Pin, Luisa Vicari Local Principal Investigator: Giorgio Moschovitis

University Hospital Geneva: Georg Ehret, Hervé Gallet, Elise Guillermet, Francois Lazeyras, Karl-Olof Lovblad, Patrick Perret, Philippe Tavel, Cheryl Teres. Local Principal Investigator: Dipen Shah

University Hospital Lausanne: Nathalie Lauriers, Marie Méan, Sandrine Salzmann. Local Principal Investigator: Jürg Schläpfer

Bürgerspital Solothurn: Andrea Grêt, Jan Novak, Sandra Vitelli. Local Principal Investigator: Frank-Peter Stephan

Ente Ospedaliero Cantonale Bellinzona: Jane Frangi-Kultalahti, Augusto Gallino. Local Principal Investigator: Marcello Di Valentino

University of Zurich/University Hospital Zurich: Fabienne Witassek, Matthias Schwenkglenks.

Medical Image Analysis Center AG Basel: Jens Würfel (Head), Anna Altermatt, Michael Amann, Petra Huber, Esther Ruberte, Tim Sinnecker, Vanessa Zuber.

Clinical Trial Unit Basel: Michael Coslovsky (Head), Pascal Benkert, Gilles Dutilh, Milica Markovic, Pia Neuschwander, Patrick Simon

Schiller AG Baar: Ramun Schmid

# References

1. Schulman S, Kearon C. Definition of major bleeding in clinical investigations of antihemostatic medicinal products in non-surgical patients. *J Thromb Haemost.* 2005;3(4):692-694.
